# The Benefits or Otherwise of Managing Malaria Cases with or without Laboratory Diagnosis: The Experience in a District Hospital in Ghana

**DOI:** 10.1371/journal.pone.0058107

**Published:** 2013-03-07

**Authors:** Kingsley Osei-Kwakye, Kwaku Poku Asante, Emmanuel Mahama, Stephen Apanga, Ruth Owusu, Evans Kwara, George Adjei, Livesy Abokyi, Enuameh Yeetey, David Kwame Dosoo, Damien Punguyire, Seth Owusu-Agyei

**Affiliations:** 1 Kintampo Health Research Centre, Ghana Health Service, Ministry of Health, Kintampo, Ghana; 2 Disease Control Department, London School of Hygiene and Tropical Medicine, London, United Kingdom; 3 Kintampo Municipal Hospital, Ghana Health Service, Ministry of Health, Kintampo, Ghana; Universidade Federal do Acre (Federal University of Acre), Brazil

## Abstract

**Background:**

This study was conducted at the Kintampo Municipal Hospital in Ghana to determine whether there was any benefit (or otherwise) in basing the management of cases of suspected malaria solely on laboratory confirmation (microscopy or by RDT) as compared with presumptive diagnosis.

**Method:**

Children under five years who reported at the Out-Patient Department of the Hospital with axillary temperature ≥37.5°C or with a 48 hr history of fever were enrolled and had malaria microscopy and RDT performed. The attending clinician was blinded from laboratory results unless a request for these tests had been made earlier. Diagnosis of malaria was based on three main methods: presumptive or microscopy and/or RDT. Cost implication for adopting laboratory diagnosis or not was determined to inform malaria control programmes.

**Results:**

In total, 936 children were enrolled in the study. Proportions of malaria diagnosed presumptively, by RDT and microscopy were 73.6% (689/936), 66.0% (618/936) and 43.2% (404/936) respectively. Over 50% (170/318) of the children who were RDT negative and 60% (321/532) who were microscopy negative were treated for malaria when presumptive diagnoses were used. Comparing the methods of diagnoses, the cost of malaria treatment could have been reduced by 24% and 46% in the RDT and microscopy groups respectively; the reduction was greater in the dry season (43% vs. 50%) compared with the wet season (20% vs. 45%) for the RDT and microscopy confirmed cases respectively.

**Discussion/Conclusion:**

Over-diagnosis of malaria was prevalent in Kintampo during the period of the study. Though the use of RDT for diagnosis of malaria might have improved the quality of care for children, it appeared not to have a cost saving effect on the management of children with suspected malaria. Further research may be needed to confirm this.

## Introduction

Malaria is a disease with a significant socio-economic impact on countries in the developing world especially in sub-Saharan Africa. [1]. Over-diagnosis and treatment of malaria is a common feature in many health facilities in sub-Saharan Africa due to the use of IMCI guidelines in the management of children under five years with fever [2], [3], [4], [5], [6]. In Ghana, 30–40% of outpatient visits at health facilities and 25% of deaths in children under five are attributed to malaria [7]. Not all of such patients are tested for malaria. Majority of the health facilities especially health centres are not equipped to carry out laboratory investigations. Even in well equipped health institutions malaria microscopy is not done for many febrile illnesses [8], [9].

Some studies have shown that more than 50% of patients who were microscopy negative for malaria were treated for the disease [10], [11]. Presumptive treatment for malaria was common in the era of cheap drugs like chloroquine and sulphadoxine-pyrimethamine (SP). However with increased resistance to those drugs and the introduction of the much more expensive artemesinin based combination therapy (ACT) [11], [12], [13], presumptive treatment of malaria has to be re-considered carefully [14], [15], [16], [17].

The ease of operation and portability of malaria rapid diagnostic tests (RDTs) as compared with that of microscopy [17], [18], [19] make it possible for them to be deployed in remote areas as well as in primary health care settings. In 2009, a combination of RDT or microscopy with ACTs was found to potentially improve the diagnosis and management of malaria cases, reduce the wastage of anti-malarial drugs and prevent resistance to antimalarials [11], [20].In settings with limited resources, evidence-based decision-making and prioritization is paramount. Restricting ACT to RDT/Microscopy positive cases alone could reduce the number of children with fever receiving ACTs by more than 50% [10], [11]. This means that the costs incurred by programme managers on treatment could easily be halved.

There have been some opposing views on whether it is probably time for changes in current guidelines recommending that African children with fever should be managed presumptively for malaria. While English et al., (2009) have expressed reservations with any attempt to introduce policy changes seeking to change presumptive treatment of malaria in favour of laboratory confirmed diagnosis and treatment [21], D'Acremont et al. (2009) have stated that the presumptive management of fevers with antimalarials currently may no longer be safe [14].

With these varied opinions in mind, we investigated the benefit or otherwise in restricting the use of ACTs to cases of malaria diagnosed by RDT/Microscopy alone compared with those presumptively diagnosed for children less than five years of age reporting to the OPD of the Kintampo Municipal Hospital. We believe that the results from this study will contribute to narrowing the knowledge gaps in the advantages or otherwise of confirmatory diagnostics (microscopy and RDTs) in the management of malaria.

## Methods

This was a cross-sectional study spanning the period from January 2009 to February 2010. It was an all-year round study that allowed for seasonality comparisons.

### Study area

The study was conducted at the Kintampo Municipal Hospital in the Kintampo North Municipality of the Brong-Ahafo Region of Ghana, which has a resident population of about 75,000 people. The municipality is located within the forest-savannah transitional ecological zone of Ghana.

The rainy season in the study area occurs between April and November each year with an average rainfall of 1250 mm per annum and mean monthly temperatures between 18°C and 38°C. The area is holoendemic in terms of malaria transmission with a parasite prevalence of more than 50% among asymptomatic children less than 10 years of age [22]. The annual entomological inoculation rate is 269 infective bites per person per year. Malaria transmission occurs perennially and the major vectors are *Anopheles gambiae* and *Anopheles funestus* with slightly more than a quarter of children under five using bed nets [22]. Studies carried out among children less than five years of age in the Kintampo area showed that children on the average could suffer up to seven (7) clinical episodes of malaria in a year [22].

Facilities for malaria microscopy are usually available at the hospital and the private clinics while RDTs are mainly used at the peripheral clinics. The community chemical shops are usually the first point of seeking medical care in the community and malaria diagnosis in the shops is mainly presumptive [23]. Currently, Artesunate-amodiaquine is the first line drug for the treatment of uncomplicated malaria in Ghana.

The municipal hospital is the referral point for the 13 Community–Based Health Planning and Service (CHPS) compounds, three (3) health centres at the sub-district levels and four private clinics in the municipality.

### Sample Size and Sampling

Using morbidity data for 2006 from the hospital OPD, a sample size of 845 children was calculated which enabled the study to have 90% power with 95% confidence. The sample size calculation was done using Stata 8.2.

#### Study procedure

Prior to the start of the study, laboratory staff on the project were trained on the correct use of the Parascreen® RDT [24] kit as well as standard preparation of blood smears for microscopy. Parascreen® is a rapid qualitative two site sandwich immunoassay for the detection of *P.falciparum* specific histidine rich protein-2(Pf.HRP-2) and Pan malaria specific Plasmodium Lactate Dehydrogenase (pLDH). The sensitivity and specificity ascribed to the test are 100% for both malaria positive and malaria negative samples [1].

Inclusion criteria for the study were children below five years of age reporting to the Out-Patient Department of the Hospital with fever (i.e. axillary temperature ≥37.5°C) or with a history of fever within the previous 48 hours. Children five years of age and above and any child admitted to the hospital with severe disease were excluded from the study.

Project staff who were stationed at the OPD identified potential study participants who were children already seen and managed by a clinician. A finger-prick blood sample (approximately 1 ml) was taken from the child by a trained laboratory technician using sterile procedures for preparation of a blood smear for microscopy examination and an RDT to confirm the presumptive diagnosis made by the clinician. The smears were independently read by two microscopists who were blinded to the results of the RDT as well as the diagnosis (es) made by the clinicians. If there was any discordance between the results of the two readers, a third and most experienced microscopist read the slide the third time, the agreement between the third reader and any of the earlier two was accepted as the final. Any asexual *Plasmodium falciparum* parasites identified were counted against 200 white blood cells. A smear was declared negative if no parasites were found after examining 100 high power fields. The parasite density was determined from the positive smears. Laboratory results from any of the tests mentioned above were made available to the clinician only upon request. No attempt was made by the study to change the treatment practices at the hospital at that time (which in most cases was presumptive).

At the time of the study children were treated according to the national IMCI guidelines which included presumptive treatment with artesunate amodiaquine and followed up. However, if a child came back unwell, the laboratory results including the blood slide results were made available to the clinician.

### Data management

All study forms were checked by the study coordinator for completeness and consistency prior to submission for data entry. Data was double entered independently into Microsoft Access database and verified. Consistency and range checks were also done and problems identified were resolved.

### Statistical analyses

The cleaned dataset was analyzed using appropriate tests in Stata 11.0. Socio-demographic characteristics of study participants that were categorical variables were summarized into proportions, while quantitative variables such as MUAC were summarized into means together with their standard deviations. The level of agreement between microscopy and RDT was estimated using the Kappa Statistic.

#### Cost of treatment relative to diagnostic methods

The study also sought to determine any differences in the cost of malaria treatment based solely on presumptive diagnosis or by laboratory confirmation. Various cost scenarios were evaluated: one was the total cost of anti-malarial treatment prescribed for subjects for whom a presumptive diagnosis of malaria had been made. This cost covered only the costs of ACTs prescribed and did not include the cost of services provided. Similar costs were calculated for subjects who were diagnosed as positive for malaria by the other two methods of diagnosis – the “Cost of malaria treatment”. Another cost was calculated separately for each group of subjects who had a positive diagnosis of malaria by either of the two laboratory diagnostic methods. This was done for each child in each group by adding the cost of antimalarials prescribed for that child to the cost of the diagnostic method – $1.00 for each RDT and $2.50 for each microscopy done (termed “Total cost of malaria treatment”). The unit cost of malaria microscopy used in the analysis was based on the cost of malaria microscopy under the Mutual Health Insurance Scheme in Ghana at the time of the study. In the second cost scenario, the two types of cost were each calculated per subject for the three diagnostic methods. Both cost scenarios were assessed separately for the wet and dry seasons. The cost of antibiotics was not included in the data analysis.

#### Ethical considerations

The study received ethical clearance from the institutional ethics committee of the Kintampo Health Research Centre (KHRC), Ghana Health Service (GHS). Mothers/caretakers voluntarily signed or thumb- printed an informed consent form after the study was fully explained to them before their children were enrolled in the study. Data was stored in locked cabinets to ensure participant confidentiality, and was only accessible to investigators and permitted members of the study team. Participants were only identified with a unique study code.

## Results

### Baseline characteristics

Nine hundred and forty (940) caregivers were contacted to participate in the study out of which 936 (99.6%) consented to participate and their children were enrolled into the study. All these children had both microscopy and RDT results for comparison with the study clinicians' diagnoses and were used for the data analysis. Table 1 shows the general characteristics of the children in the study.

**Table 1:** Baseline demographic characteristics study participants.

(N = 936) Categories	Age (months)			
	0–11	12–23	24–59	Total
	n (%)	n (%)	n (%)	n (%)
**Sex**				
Males	132 (57.1)	131(50)	230(51.9)	493(52.7)
Females	99 (42.9)	131(50)	213(48.1)	443(47.3)
**Total**	**231 (24.7)**	**262 (28)**	**443 (47.3)**	**936(100)**
**Weight(kg):** mean (SD)	7.5(1.6)	9.6(1.5)	12.7(2.4)	10.6(3.0)
**Weight-for-age z-score** Mean ± SD (95% CI)	−0.28±1.31 (−0.45,−0.11)	−0.78±1.26 (0.28, 1.00)	−0.98±1.24 (−1.03, −0.42)	−0.75±1.30 (−0.83, −0.67)
**Height-for-age z-score** Mean ± SD (95% CI)	0.64±2.36 (0.28, 1.00)	−0.58±2.17 (−0.88, −0.27)	−1.01±1.41 (−1.16, −0.86)	−0.50±1.99 (−0.65, −0.36)
**Weight-for-height z-score** Mean ± SD (95% CI)	−0.73±1.99 (−1.03,−0.42)	−0.74±1.70 (−0.98, −0.51)	−0.63±1.60 (−0.80, −0.46)	−0.69±1.73 (−0.81, −0.56)
**MUAC** * **(cm) (n = 920)** Mean (SD)	14.1 (1.4)	14.3 (1.2)	15.1 (1.3)	14.7 (1.4)

* **MUAC- Mid Upper Arm Circumference.**

Children recruited into the study were between 1 month and 59 months of age (mean = 24 months) with 52.7% of them being males. Children between the ages of 24 months and 59 months constituted the largest proportion of study participants. There was not much difference between the proportions of children in the 0–11 month and 12–23 month age groups (24.7% vs. 28.0%). The Z-scores were based on the WHO 2005 Standard population.

Table 2 shows the various symptoms that the children presented with at the hospital. The most prevalent symptoms on presentation were poor appetite, cough, vomiting and diarrhea. Axillary temperatures recorded ranged between 35.7°C and 40.7°C (mean = 37.6°C).

**Table 2:** Clinical features of respondents (symptoms at presentation).

Symptoms	n (%)
Poor Appetite	555 (59.5)
Cough	464 (49.8)
Vomiting	429 (46.0)
Diarrhoea	298 (32.0)
Irritability	39 (4.2)
Fast breathing	18 (1.9)
Difficulty in breathing	6 (0.6)

### Characterization of diagnosis based on presumption, microscopy or RDT

**Figure 1:**
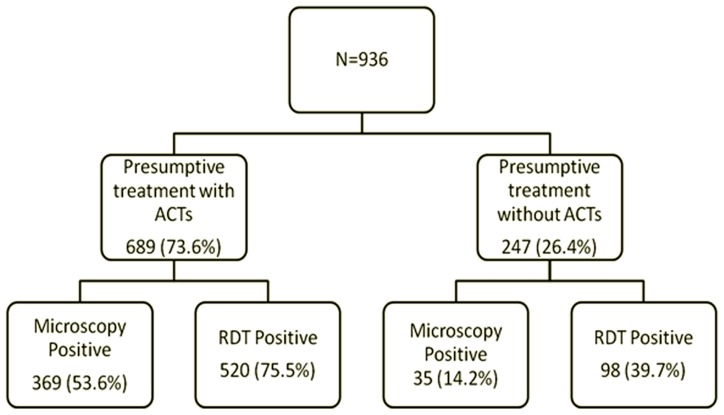
Over-diagnosis and missed diagnosis of Malaria.

The proportions of malaria which were diagnosed presumptively, by RDT and by microscopy were 73.6% (689/936), 66.0% (618/936) and 43.2% (404/936) respectively. Figure 1 shows the proportions of presumptively treated malaria cases which were diagnosed as having the disease by the two confirmatory methods. Microscopy revealed that just a little over half (53.6%) of the children who were presumptively diagnosed with malaria and were treated with ACTs, were positive for the disease. With RDT, 75.5% of the same patients had malaria. Microscopy and RDT identified 14.2% and 39.7% respectively of the children as being positive for malaria even though they were presumptively diagnosed as non-malaria cases and therefore not treated with ACTs.

**Figure 2:**
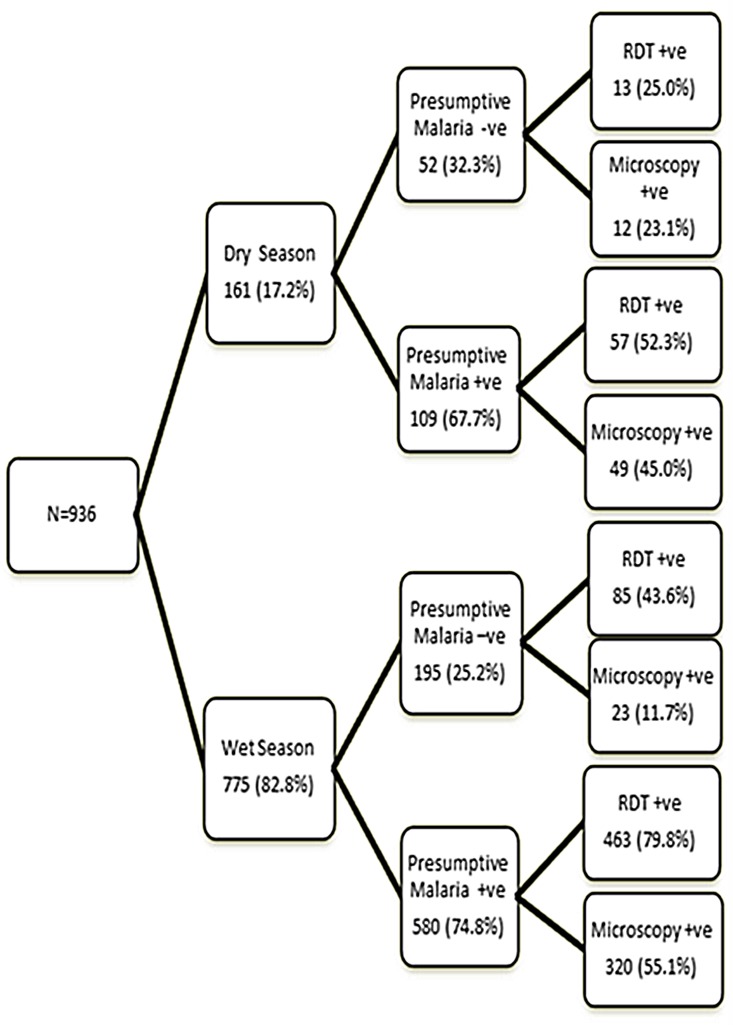
Seasonal variation of Malaria diagnosis by the three methods.

The data was disaggregated by seasonality (Figure 2). Of the 936 children enrolled into the study, 82.8% and 17.2% were enrolled in the wet and dry seasons respectively. About three quarters of children were presumptively diagnosed with malaria in the wet season. Almost eighty percent (80%) of the presumptively diagnosed malaria cases in the wet season were positively confirmed as such by RDT in contrast to microscopy that confirmed just a little over half of them. In the dry season, microscopy and RDT confirmed almost similar proportions of the presumptively diagnosed malaria cases.

Only one percent (1%) of children diagnosed as non-malaria cases by RDT in the dry season was identified as positive for the disease by microscopy. Almost a quarter (23.8%) of study subjects diagnosed as malaria cases by RDT were not confirmed by microscopy (Table 3).

**Table 3:** Comparison between the laboratory diagnostic methods by seasons.

	Microscopy	
Wet Season	Positive n (%)	Negative n (%)
RDT Positive	337 (43.5)	211 (27.2)
RDT Negative	6 (0.8)	221 (28.5)
**Dry Season**		
RDT Positive	58 (36.0)	12 (7.5)
RDT Negative	3 (1.9)	88 (54.7)
**Overall**		
RDT Positive	395 (42.2)	223 (23.8)
RDT Negative	9 (1.0)	309 (33.0)

### Sensitivity and specificity of Presumptive diagnosis and RDT

The sensitivity of the presumptive method in diagnosing malaria in the wet and dry seasons was 93.3% and 80.3% respectively (Table 4). The RDT showed both a higher sensitivity and specificity for diagnosing malaria as compared to presumptive diagnosis though both methods were not highly specific in diagnosing the disease. The sensitivities of both presumptive and RDT in diagnosing malaria decreased in the dry season with the decrease for the presumptive method (from 93.3% to 80.3%) greater than that for the RDT (98.3% to 95.1%). While there was virtually no decrease in the specificity of the presumptive method in diagnosing malaria between the two seasons, that of the RDT rather increased in the dry season (from 51.2% to 88.0%). Overall, using microscopy as the gold standard, the sensitivity and specificity of the RDT used were 97.8% (95% CI 95.8–99.0) and 58.1% (95% CI 53.8–62.3) respectively. Children who were presumptively diagnosed as non-malaria cases had a lower mean parasite density [5613 (95% CI 2643, 11 918)] compared to children presumptively diagnosed as malaria cases [38 310 (95% CI 31 270, 46 936)]

**Table 4:** Sensitivity and Specificity of Presumptive diagnosis and RDT diagnosis of malaria by seasons using Microscopy as the gold standard (with 95% CI).

	Sensitivity (%) (95% CI)	Specificity (%) (95% CI)	PPV (%) (95% CI)	NPV (%) (95% CI)
	**Presumptive vs. Microscopy**			
**WET**	93.3 (90.1–95.7)	39.8 (35.2–44.6)	55.2 (51.0–59.3)	88.2 (82.8–92.4)
**DRY**	80.3 (68.2–89.4)	39.0 (29.4–49.3)	44.6 (35.1–54.3)	76.5 (62.5–87.2)
**OVERALL**	91.3 (88.2–93.4)	39.7 (35.5–44.0)	53.5 (49.7–57.3)	85.8 (80.8–89.9)
	**RDT vs. Microscopy**			
**WET**	98.3 (96.2–99.4)	51.2 (46.3–56.0)	61.5 (57.3–65.6)	97.4 [94.3–99.0)
**DRY**	95.1 (86.3–99.0]	88.0 (80.0–93.6)	82.9 (72.0–90.8)	96.7 [90.7–99.3)
**OVERALL**	97.8 (95.8–99.0)	58.1 (53.8–62.3)	63.9 (60.0–67.7)	97.2 [94.7–98.7)

Table 5 shows the level of agreement between various diagnostic methods employed in the study. The highest level of agreement was between microcopy and RDT (75.2%).

**Table 5:** Level of agreement between various malaria diagnostic methods.

Method of Diagnosis (N = 936)	% Agreement	Kappa statistic	p-value
Microscopy and Presumptive malaria	62.1	0.29	P<0.001
RDT and Presumptive malaria	71.5	0.33	P<0.001
Microscopy and RDT	75.2	0.53	P<0.001

If the diagnosis of malaria had to be confirmed by any of the laboratory methods before ACTs were given, the reduction in over-diagnosis using RDTs would have been 4.1% and 24.2% (diff 20.1%, CI 13.3%–26.9%, p<0.001) in the wet and dry seasons respectively. With microscopy, the reduction would have been 30.5% and 29.8% (diff 0.7%, CI −7.1%–8.5%, p = 0.86) respectively. Overall, over-diagnosis of malaria would have been reduced by 7.6% (CI 3.4%–11.7%, p<0.001) and 30.5% (CI 26.2%–34.7%, p<0.001) using RDT and microscopy respectively.

Tables 6 show the results generated for the various scenarios mentioned above. The cost of malaria treatment in the RDT and microscopy diagnosed groups were lower by 24% and 46% respectively, compared to that incurred for the presumptive group ($528.13). The reductions in costs were greater in the dry season (48% vs. 56%) than in the wet season (20% vs. 45%) for the RDT and microscopy groups respectively. However in terms of the total cost of treatment, RDT and microscopy were more expensive than presumptive treatment (Table 6). The cost of malaria treatment per subject was the same for all three methods except for a very marginal difference in the dry season. However, the total cost of treatment was 2.3 and 4.2 times more for the RDT and microscopy respectively as compared to the presumptive method (Table 6).

**Table 6:** Total malaria treatment costs.

	Total Cost for all subjects		Per patient cost	
Method of diagnosis	Cost of malaria treatment in USD (95% CI)	Total cost of malaria treatment in USD** (95% CI)	Cost of malaria treatment in USD (95% CI)	Total cost of malaria treatment in USD** (95% CI)
**WET SEASON**				
PRESUMPTIVE (N = 580)	452.29 (429.27–475.32)	452.29 (429.27–475.32)	0.78 (0.74–0.82)	0.78 (0.74–0.82)
RDT (N = 463)	363.30 (341.88–384.72)	826.30 (804.88–847.72)	0.78 (0.74–0.83)	1.78 (1.74–1.83)
MICROSCOPY (N = 320)	249.48 (232.14–266.82)	1049.48 (1032.14–1066.83)	0.78 (0.73–0.83)	3.28 (3.23–3.33)
**DRY SEASON**				
PRESUMPTIVE (N = 109)	75.84 (74.46–77.22)	75.84 (74.46–77.22)	0.70 (0.68–0.71)	0.70 (0.68–0.71)
RDT (N = 57)	39.25 (37.95–40.55)	96.25 (94.95–97.55)	0.69 (0.67–0.71)	1.69 (1.67–1.71)
MICROSCOPY (N = 49)	33.65 (32.34–34.96)	156.15 (154.84–157.46)	0.69 (0.66–0.71)	3.19 (3.16–3.21)
**OVERALL**				
PRESUMPTIVE (N = 689)	528.13 (505.03–551.24)	528.13 (505.03–551.24)	0.77 (0.73–0.80)	0.77 (0.73–0.80)
RDT (N = 520)	402.55 (381.06–424.04)	922.55 (901.06–944.04)	0.77 (0.73–0.82)	1.77 (1.73–1.82)
MICROSCOPY (N = 369)	283.13 (265.72–300.55)	1205.63 (1188.21–1223.05)	0.77 (0.72–0.81)	3.27 (3.22–3.31)

** **Cost of anti-malarial treatment+cost of diagnostic method.**

## Discussion

This study showed that the highest frequency of treatment for malaria (73.6%) was recorded by the presumptive method of diagnosis followed by RDT (66%) and microscopy (43.2%). Results from this study are similar to those in Tanzania which showed that over 50% of patients for whom antimalarials were prescribed may not have had the disease [2], [3]. As shown in Figure 1, close to half of the children who were presumptively treated for malaria were microscopy negative. Over-diagnosis of malaria and consequent treatment is a public health problem because it leads to increased reporting of the malaria burden with resultant misallocation of resources to manage the disease, wastage of antimalarials and increased threat of resistance to ACTs. It also results in increased attendance to health facilities due to poor response to treatment (potential misdiagnosis of serious non-malarial infections) and consequent increased workload on the already under staffed and inadequately resourced health facilities [6], [10]. With major concerns about parasite resistance development to the ACTs and the high costs of the ACTs, the judicious use of these drugs needs to be given high priority.

The high levels of agreement recorded between RDT and microscopy as diagnostic methods means in transmission areas comparable to ours, one of these diagnostics methods if available, is sufficient as a diagnostic method for malaria. The World Health Organization (WHO) recommends laboratory confirmation (either by microscopy or RDT) of all suspected malaria cases before treatment is commenced and that presumptive treatment should only be considered where such confirmation cannot be done [1]. Of the two laboratory methods, RDTs appear to be the method receiving the more prominent attention as they are perceived as having the potential to make a significant impact on improving the diagnosis of malaria. This is because RDTs produce quicker results; do not require any high level of skills to perform them, as opposed to microscopy, which requires more time and reagents, equipment and well-trained/dedicated staff to produce quality results [6], [10], [25].

In this study, the RDT had a high sensitivity (97.7%) but a rather low specificity (58.1%) for detecting malaria. In terms of diagnosing malaria there was moderate agreement between RDT and microscopy (Kappa = 0.53) [4]. The Parascreen® RDT used in this study satisfies one of the criteria for a useful diagnostic tool for RDTs with its high sensitivity (97.7%) but not for specificity. In spite of its low overall specificity, the marked increase in its specificity from 51.2% in the rainy season to 88.0% in the dry season showed that it could be a valuable tool to use to improve the diagnosis of malaria during the dry period, especially as there was not much decrease in its sensitivity during the same period. This means that the specificity of the RDT is critical in the dry season when the prevalence of malaria is relatively lower. On the other hand, a high sensitivity of the RDT will be required in the wet season when the malaria prevalence is very high.

As earlier stated, almost a quarter of study subjects diagnosed as malaria cases by RDT were not confirmed by microscopy (Table 3). This was likely due to prior treatment with antimalarials with consequent clearing of parasitaemia and persistence of HRP2 antigenemia [26].

With regards to the cost of treatment in the various groups, overall the cost of treatment (per subject) (Table 6) did not differ among the three groups. The total cost of treatment was higher in the RDT and microscopy groups even though fewer subjects were diagnosed with malaria in those groups than in the presumptive group. These results seem to suggest that RDTs are not cost-saving when used in the management of malaria in children less than five years, a result that is similar to that of Msellem et al. (2009), which found that cost-reduction using RDTs was not achieved among patients under 5 years but rather among those who were 15 years and above [27]. The low positive predictive value and specificity of the RDT lends possible credence to this negative cost-saving effect though the latter property of the RDT contrasts sharply with the 100% for both sensitivity and specificity recorded in a study by the manufacturer [24]. The cost of antibiotics was not included in this study. It is likely that children who had a false negative RDT were treated with antibiotics, however, this cost may not be lower than the cost of over-treatment of malaria with ACTs as suggested by Shillcott et al [28].

Notwithstanding the high total cost of malaria treatment in the RDT group, a potential limiting factor for its use, the RDT can still be said to be an effective tool in reducing the over-diagnosis of malaria and the consequent use of ACTs in non-malaria cases, due to its high negative predictive value. The quality of care of such children will therefore be improved [14], [15], [17], [29].

### Limitations

One limitation of the study was that since it was a cross-sectional study, there was no follow up of the subjects. It was therefore not possible to ascertain the clinical outcomes of the children who were presumptively treated for malaria (especially those for whom the diagnosis of malaria was not confirmed by RDT or microscopy) and those who were not treated for malaria even though they had been diagnosed as having the disease by the two laboratory methods.

Another possible limitation that since the study was conducted only in children less than five years of age and not in participants across all age groups, the possible cost-saving effect of the RDT could not be determined conclusively.
